# A new three-dimensional patient-specific cutting guide for opening wedge high tibial osteotomy based on ct scan: preliminary in vitro results

**DOI:** 10.1186/s40634-023-00647-3

**Published:** 2023-08-09

**Authors:** Federica Rosso, Roberto Rossi, Philippe Neyret, Robert Śmigielski, Jacques Menetrey, Davide Edoardo Bonasia, Sandro Franco Fucentese

**Affiliations:** 1 Department of Orthopaedics and Traumatology, AO Ordine Mauriziano Hospital, Largo Turati 62, 10128 Turin, Italy; 2 Infirmerie Protestante, 3 Rue Penthod, 69300 Caluire, France; 3 Orthopaedics Department, Reem Hospital, Abu Dhabi, UAE; 4 Orthopaedics and Sports Medicine Department, Life Institute Biological Treatment Center Lead, Grzybowska 43A/U8, Warsaw, Poland; 5 Centre de Medecine du Sport Et de L’Exercice, Hirslanden Clinique La Colline, Genéve, Switzerland; 6 Department of Orthopedics, Balgrist University Hospital, University of Zurich, Forchstrasse 340, 8008 Zurich, Switzerland

**Keywords:** Patient-specific, PSI, High tibial osteotomy, HTO, Opening wedge, Accuracy

## Abstract

**Purpose:**

The aim of this study was to evaluate the accuracy of a patient-specific cutting guide on both coronal and sagittal alignment compared to the pre-operative planning in OWHTO.

**Methods:**

Twelve OWHTO on 6 cadaveric specimens were performed by 3 experienced knee surgeons using patient-specific cutting guides based on 3D pre-operative planning. Since the specimens had no major deformities, a fixed correction of 6° on the left and 10° on the right legs were carried out to simulate different scenarios. A pre-operative and post-OWHTO 3D CT scans were performed, and images were superimposed using the dedicated 3D planning software to align their reference axes. A pre-operative planning was performed considering both Medial Proximal Tibial Angle (MPTA) and Posterior Tibial Slope (PTS), and a patient-specific cutting guide was produced. Planned and post-OWHTO MPTA and PTS were evaluated (mean and standard deviation), and Pearson’s correlation coefficient was calculated to assess precision and accuracy of the whole treatment.

**Results:**

A mean correction of 6,1° (SD 1,9°) and 1,2° (SD 1°) was obtained respectively in the coronal plane (MPTA) and in the sagittal plane (PTS). The average difference between planned and post-OWHTO MPTA and PTS was respectively 1,2° (SD 0,6°) and 1,2° (SD 1°) in the sagittal plane (PTS). Pearson’s correlation coefficient demonstrated a good accuracy of the treatment in both coronal and sagittal plane (respectively *r=*0,95 and *r=*0,86). No lateral hinge fractures were detected at the post-operative CT scan.

**Conclusion:**

OWTHO performed with the help of 3D patient specific cutting guide on cadaveric specimens demonstrated good accuracy and reliability in obtaining the planned correction. In vivo studies are necessary to confirm these results and evaluate cost-effectiveness of this system.

**Level of evidence:**

Level IV cadaveric study.

## Introduction

Opening Wedge High Tibial Osteotomy (OWHTO) is a valid treatment for medial compartment arthritis/overload in young patients with a varus aligned knee [1, 2]. The aim of OWHTO is to correct the limb malalignment on the coronal plane, but some effect on posterior tibial slope (PTS) should be expected. Particularly, Noyes et al demonstrated that medial OWHTO increases the tibial slope only if the anteromedial gap is equal to the posteromedial gap, whereas the slope does not change if the anteromedial gap is smaller than the posteromedial one [3]. Furthermore, it has been recently confirmed that not all the varus deformities should be corrected on the tibia only, because it can produce an excessive increase of the medial Mechanical Proximal Tibial Angle (MPTA) which may result in pathological lateral inclination of the joint line, increased cartilage shear stress and possible inferior clinical outcome [2, 4]. Historically, the goal of OWHTO was to shift the mechanical axis from the medial to the lateral side of the knee, aiming at the Fujisawa point, which was located on the tibial plateau at 62.5% of the medial-lateral width [5]. However, in the last years different authors demonstrated that correcting up to Fujisawa point may lead to a greater risk for lateral compartment damage, and a correction to 55% of the tibial width, which represent the apex of the lateral tibial spine, may be a good compromise to avoid excessive under-correction or lateral compartment damage [6].

In this scenario, an accurate pre-operative planning with identification of the correct osteotomy site and amount of correction on both coronal and sagittal plane is mandatory [7, 8]. Different studies confirmed that accuracy of correction relative to preoperative planning is strictly associated to HTO outcomes [9, 10]. Unfortunately, conventional technique (i.e. radiopaque cable under fluoroscopy) achieves post-operative alignment within 3° of planned mechanical femoro-tibial angle (mFTA) in only 50-70% of the cases [11].

Computer-assisted surgery (CAS) may help surgeons in obtaining a better intra-operative accuracy for mechanical axis, but this technology is costly and associated to a long learning curve [12]. However, some studies reported on high rate of outliers also with CAS HTO, even if they were not associated to worse outcomes [13].

Recently 3D patient-specific cutting guides (PSCG) based on Computer Tomography (CT) scans were developed. Preliminary in vitro studies described very good accuracy for some PSCG systems on both the coronal and sagittal plane [14–17].

In this study, a new PSCG based on pre-operative CT scans for OWHTO was tested. The first aim of this study was to evaluate the accuracy of the system on both coronal and sagittal plane. Secondary aim of the study was to evaluate the number of complications related to the osteotomy (i.e. intra-articular fracture), which were supposed to be reduced with a PSCG.

## Material and methods

### Study design

Six frozen human cadavers were obtained from ScienceCare ®. Cadavers with previous surgery on the lower limb, or previous skeletal deformity, were excluded.

A total of 12 HTO were performed, 6 on the left and 6 on the right knees, by 3 experienced knee surgeons, with good experience in performing OWHTO (R.R., N.P., S.R.).

### OWHTO pre-operative planning

All specimens were scanned pre- and post-operatively with the same CT scan protocol.

To be able to simulate the realignment osteotomy (tibia and/or femur) based on the mechanical leg axis, 3D models of the corresponding bone parts must be obtained. The proximal femur, the knee, and the ankle joints were acquired in a single CT scan to avoid specimen’s movements. Different points should be considered in both clinical and experimental setting to obtain a correct CT data, and they were respected in both pre and post-operative CT scan: 1) axial resolution of 1 mm, in-plane resolution <0.4 mm, peak kilovoltage 120 kvp and tube current 135 mA; 2) Field Of View maximum 200 mm; 3) Slice Thickness (Hip, knee and ankle) of 1 mm, with slice increment of 0-5 mm; 4) hip should be included from the proximal joint space to at least ¼ of the proximal femoral shaft, with the lower conical x-ray beam limit at the lesser trochanter; 5) knee should be included from 15 cm above to 20 cm below the joint line; 6) ankle should be included from 5 cm above the joint line to the center of calcaneus (including lateral and medial). All the DICOM images were imported in a dedicated software (MyOsteotomy®, Medacta, Switzerland), and a 3D geometrical model was created for pre-operative planning. Preoperative Mechanical Proximal Tibial Angle (MPTA) and Posterior Tibial Slope (PTS) were measured to evaluate coronal and sagittal plane alignment, since an accuracy on both plane is mandatory to obtain good outcomes.

**Fig. 1:**
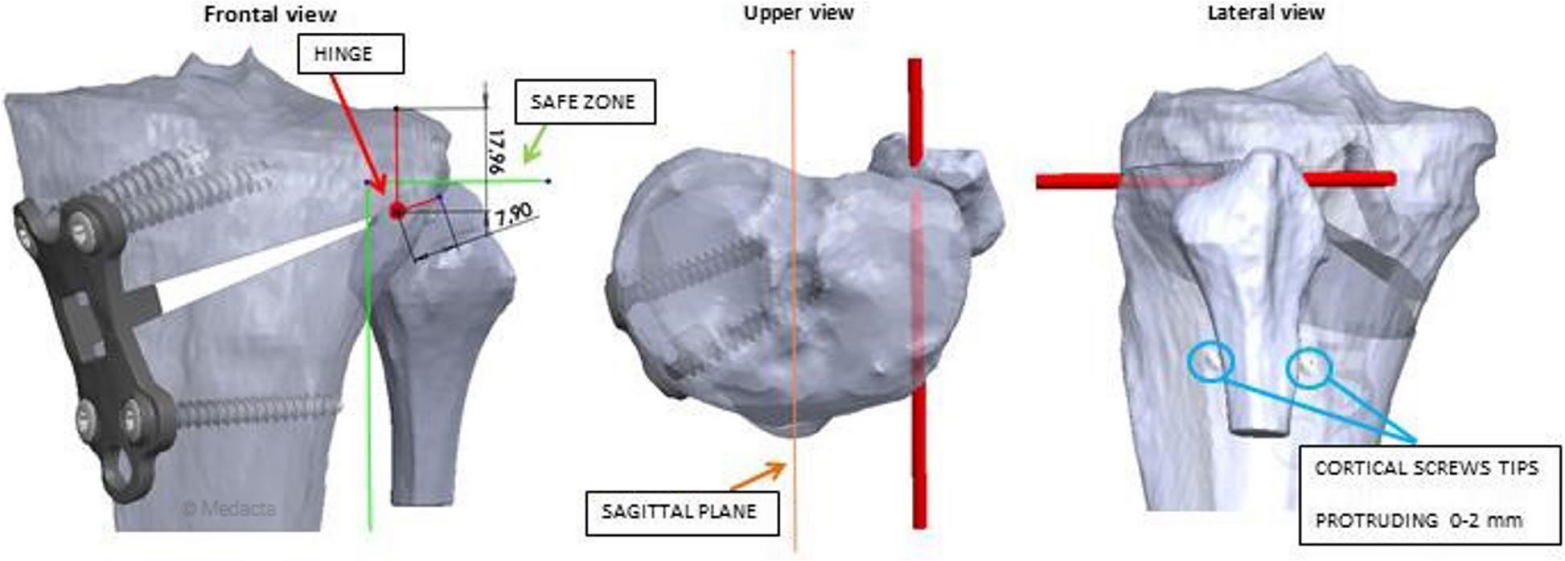
Planning of the hinge passing through the ‘safe zone’ as described by Nakamura et al [18]. The red bar shows the hinge in the frontal, upper and lateral view

**Fig. 2:**
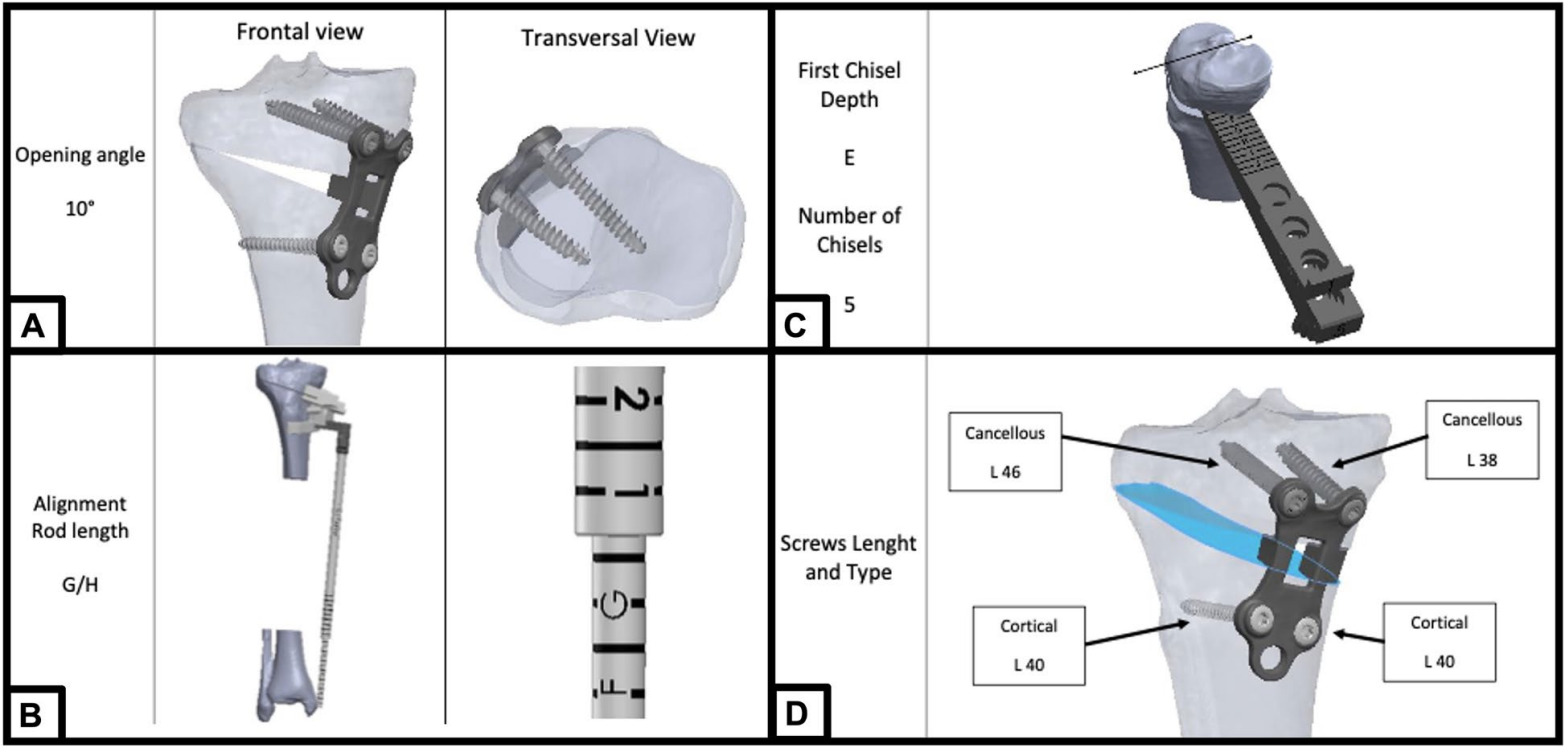
Definitive pre-operative planning of one of the cases. **A** Frontal and axial view of the plate and screws; **B**) Length and position of the alignment rod which will be reproduce intra-operatively to confirm the guide positioning; **C**) First chisel depth and number of chisels needed to obtain the planned gap opening; **D**) screw length and type

Since the specimens had no major deformities, a fixed correction of 6° on the left and 10° on the right legs were carried out to simulate different scenarios. The pre-operative planning was performed with Solidworks^®^ using the registered MyKnee^®^ procedure (Medacta, Switzerland), to calculate anatomical axes, planes and landmarks. The three involved surgeons were invited to complete the planning, checking both cutting plane position and results from OWHTO simulation. If surgeons were not satisfied from automated simulation, they could modify both planning and cutting plane position. The osteotomy was oriented in such a way that tibial slope was not affected (parallel to sagittal plane) and the hinge was planned passing through a ‘safe zone’ to avoid bone fracture propagation, identified by tibio-fibular joint area [18] (Fig. 1). The planning was then confirmed, and the surgeon received all the information to perform the osteotomy (including amount of opening, saw blade and chisel depth and number to avoid lateral hinge fracture, plate positioning and screws length) as well as the personalized cutting guide and relative proximal tibia model (Fig. 2).

### OWHTO surgical technique

**Fig. 3:**
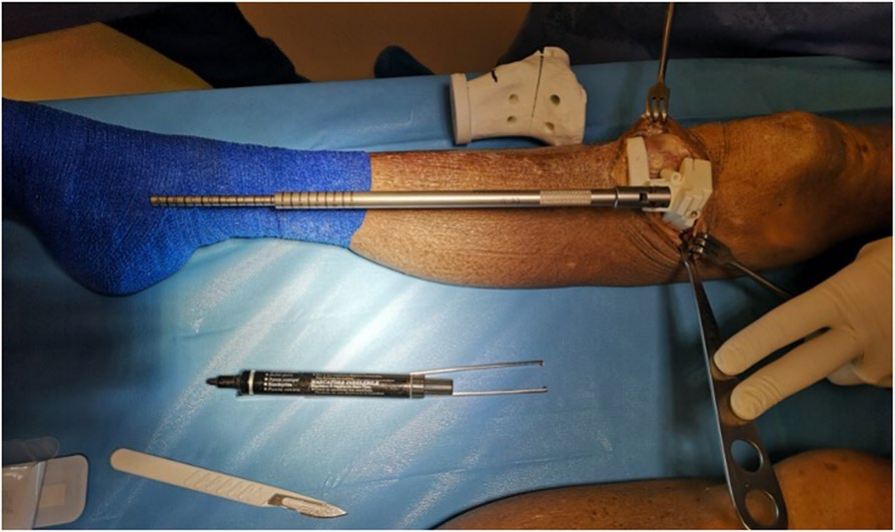
Positioning of the cutting guide. The personalized cutting guide is positioned on the antero-medial aspect of the tibia and the position is checked with the appropriate rod indicating the same lenght of the pre-operative planning

**Fig. 4:**
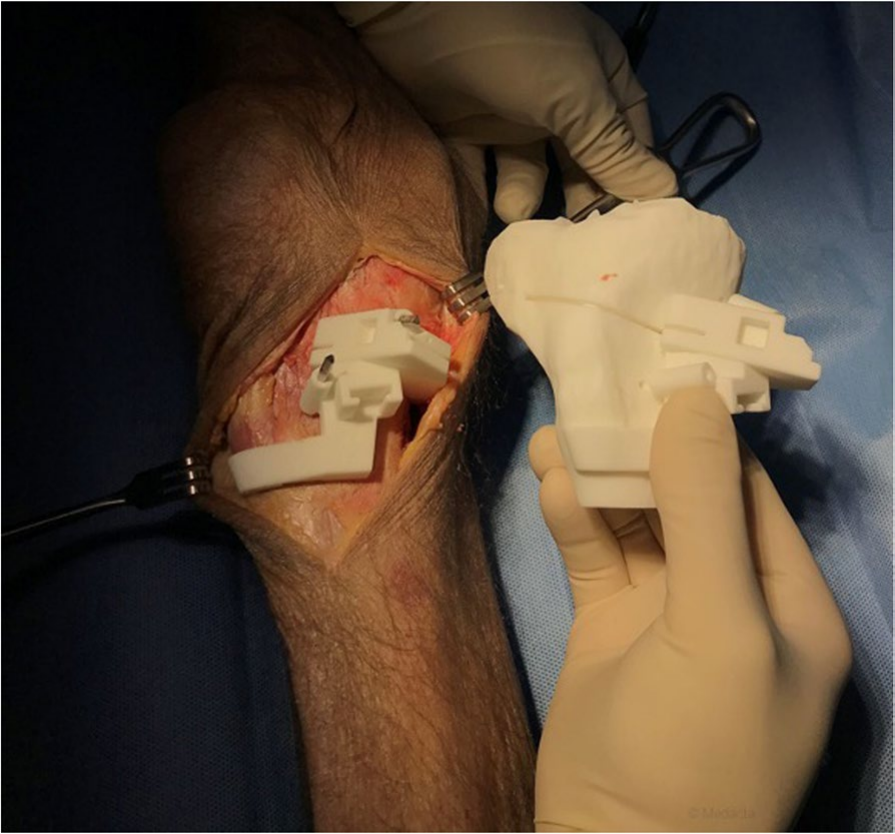
Fixation of the cutting guide. The personalized cutting guide is fixed with pins and its position is checked compared to the 3D printed model of the proximal tibia

A 5 cm incision on the anteromedial aspect of the tibia, extending from 1 cm below the medial joint line midway between the medial border of the tibial tubercle and the posteromedial border of the tibia was performed, as previously described [19]. The sartorius fascia, pes anserinus and Medial Collateral Ligament (MCL) were retracted. The personalized cutting guide was positioned on the antero-medial tibia, and the position was checked with the dedicated alignment rod (Figs. 3 and 4).

**Fig. 5:**
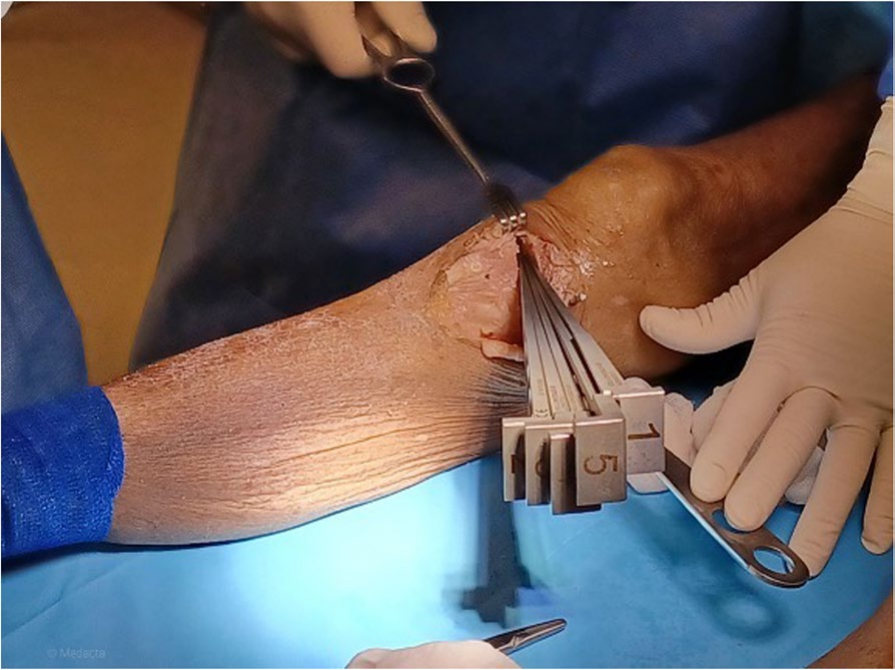
Gap opening. Intra-operative picture showing the chisels and gap opening The number of chisels correspond to the amount of gap opening and it is reported in the pre-operative planning

**Fig. 6:**
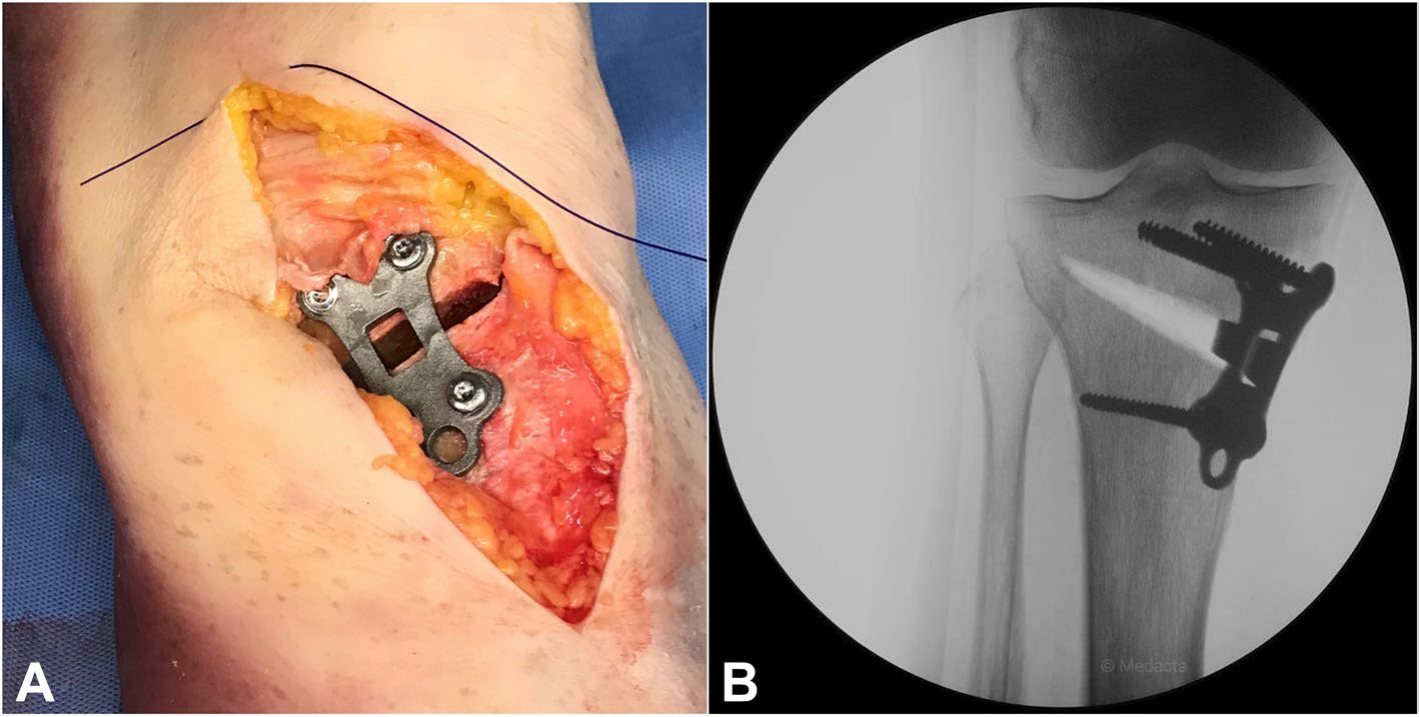
Final result. **A** The personalized plate in position. **B** Antero-posterior view of the intra-operative x-ray

When the correct position is achieved, two 3.2 mm pins are inserted to fix the cutting guide, and the k-wires can be inserted into the dedicated holes in the cutting slot to check osteotomy orientation with fluoroscopy and the dedicated alignment rod. The bone cut was performed through the cutting slot with a 1 mm saw blade, the anterior pin was removed as well as the cutting guide, leaving only the posterior pin as a reference. The osteotomy was then performed with dedicated chisels in a standard manner, being careful not to damage the lateral hinge point and to avoid unexpected crack propagation. First chisel length was indicated in the pre-operative planning, as shown in Fig. 1. Chisels are consequently numbered to obtain the appropriate gap opening, depending on the number of chisels planned (Fig. 5). Once the osteotomy is opened and the gap is as planned, the toothed plate is positioned. The tooth is custom-made to the exact dimension and shape planned. The alignment was checked intra-operatively with fluoroscopy and the dedicated rod. Also, the position of the plate against the planned position are checked mechanically. Once the correct position of the plate is verified, the screws can be inserted, starting from the posterior distal (cortical screw) and the anterior proximal (cancellous screw). After checking that the inferior osteotomy plane is well in contact with the wedge of the plate, the last two screws can be inserted. Length and position of all the screws are reported in the planning. Figure 6 shows the result.

### Registration process

**Fig. 7:**
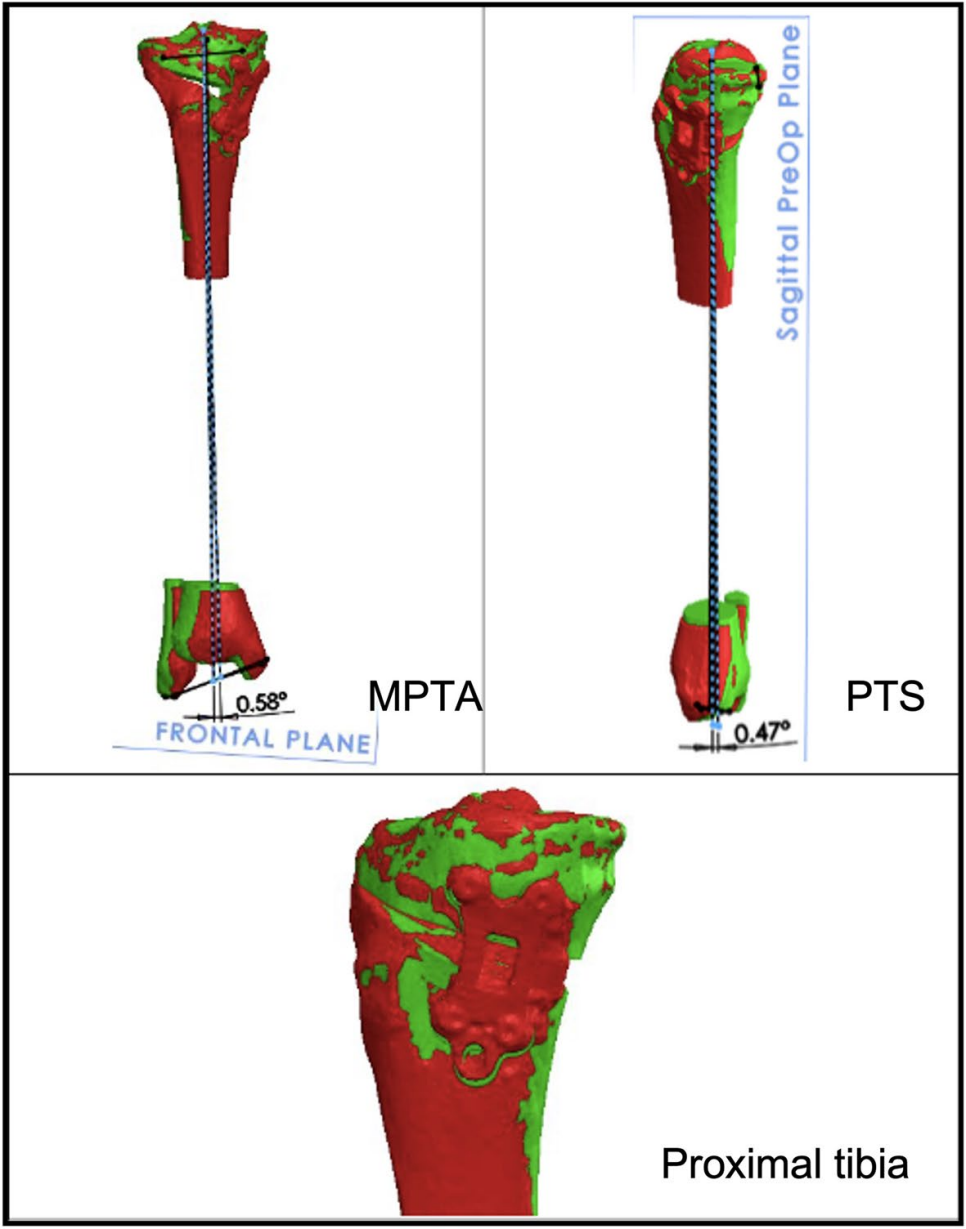
Registration process of MPTA, PTS and Proximal tibia. In green the planned model, in red the superimposed post-OWHTO model. (MPTA=Mechanical proximal tibial angle, PTS=Posterior Tibial Slope)

All the specimens underwent post-operative fluoroscopy and CT scan. To compare pre-operative and post-OWHTO CT scans, the images of the bones were aligned in the area of the tibial plateau that was not affected by the osteotomy using a best-fit algorithm in CASPA^®^ software. With this strategy, all anatomical landmarks in the proximal tibia (e.g. tibia center, lateral and medial posterior walls, tibial lateral and medial points, anterior and posterior plateau points) were exactly coincident in all scenarios (pre-operative, planned OWHTO and post-operative), thus avoiding errors in plane and axes calculation that could originate from using different anatomical landmarks (Fig. 7).

MPTA angle and PTS were compared in planned-OWHTO and post-OWHTO scenarios to assess the precision and accuracy of the treatment.

### Statistical analysis

Statistical analysis was performed between planned-OWHTO and post-OWHTO MPTA and PTS angles. Pearson’s correlation test was used to evaluate the accuracy of patient-specific cutting guide comparing planned and post-OWHTO MPTA and PTS. Significance was considered at *p<*0,05. Data were presented with mean, range, and standard deviation (SD), as well as Confidence Intervals (CI) if available. Considering a standard deviation of 1° and a clinically relevant difference of 1,5°, with α=0,05 and β=0,20, 7 samples were necessary to achieve statistical significance.

## Results

The mean pre-operative MPTA was 87,9° (SD 1,8°) and the mean pre-operative PTS was 8° (SD 3°). A mean correction of 6,1° (SD 1,9°) was obtained in the coronal plane (MPTA) and 1,2° (SD 1°) in the sagittal plane (PTS).

For all the specimens, the cutting guide was positioned on the tibial bone as planned, with a good fit, so all the osteotomies were performed according to the planning.

The mean difference between the planned-OWHTO and post-OWHTO configuration was 1,2° (SD 0,6°) on the coronal plane (MPTA) and 1,2° (SD 1°) in the sagittal plane (PTS) (Table 1).

**Table 1:** Angular values for both MPTA angle and PTS (OWHTO= Opening Wedge High Tibial Osteotomy, MPTA=Mechanical Proximal Tibial Angle, PTS=Posterior Tibial Slope)

**Patient ID**	**Pre-OWHTO**		**Planned-OWHTO**		**Post-OWHTO**		**Planned-OWHTO vs Post-OWHTO**	
	**MPTA**	**PTS**	**MPTA**	**PTS**	**MPTA**	**PTS**	**MPTA**	**PTS**
**1**	87,2	1,4	92,8	1,4	91,6	1,9	1,2	0,5
**2**	85,4	8,0	94,7	8,0	94,1	7,5	0,6	-0,5
**3**	85,9	8,9	91,0	8,9	90,8	7,7	0,3	-1,2
**4**	85,1	6,0	95,3	6,0	93,4	6,8	1,9	0,8
**5**	91,0	7,5	96,2	7,5	95,6	7,5	0,7	0,0
**6**	89,1	12,1	98,2	12,1	97,1	12,9	1,1	0,7
**7**	89,1	8,0	94,5	8,0	92,9	9,4	1,6	1,4
**8**	89,3	12,1	94,7	12,1	94,3	10,6	0,5	-1,5
**9**	89,4	7,5	94,3	7,5	93,2	9,3	1,1	1,8
**10**	88,5	5,5	97,9	5,5	95,8	4,9	2,1	-0,6
**11**	86,9	10,9	96,1	10,9	94,5	8,9	1,6	-2,0
**12**	87,9	7,7	97,3	7,7	95,2	11,3	2,2	3,6

Pearson’s correlation coefficient was used to evaluate the association between planned-OWHTO and post-OWHTO values, demonstrating a strong correlation for both MPTA and PTS (MPTA *r=*0,95, 95%CI 0,83-0,98, *p<*0,001; PTS *r=*0,86, 95%CI 0,56-0,96, *p*=0,004, Table 2).

**Table 2:** Summary of the association between planned-OWHTO and post-OWHTO values calculated with the Pearson’s correlation coefficient (OWHTO= Opening Wedge High Tibial Osteotomy, MPTA=Mechanical Proximal Tibial Angle, PTS=Posterior Tibial Slope, CI95%= Confidence Interval, SD=Standard Deviation)

**Angle measured**	**Average Planned-OWHTO (SD)**	**Average Post-OWHTO (SD)**	**Pearson Coefficient (r)**	***P*-value**	**CI95%**
**MPTA**	95,3° (2,1°)	94° (1,8°)	0,95	<0,001	0,83-0,98
***PTS***	8° (3°)	8,2° (2,9°)	0,86	0,004	0,56-0,96

No complication related to the osteotomy, such as lateral hinge fractures, were detected at the post-operative CT scan. Furthermore, all the ostomies were performed at the planned level, confirming no tibial guide malposition.

## Discussion

The most important finding of the present study is that this PSCG demonstrated good accuracy in both coronal and sagittal plane, with high correlation between planned and post-operative MPTA and PTS (respectively *r=*0,95 and *r=*0,86). The average difference between planned and post-operative MPTA was 1,2° (SD 0,6°), which can be considered acceptable. Similarly, the average difference between planned and post-OWHTO PTS was 1,2° (SD 1°), demonstrating a good control of the tibial slope, which is mandatory to achieve good outcomes after OWHTO. In all the cases the cutting guide was correctly positioned according to the digital pre-operative planning and the 3D-printed model.

OWHTO has been reported to offer good long-term outcomes if performed with correct indications and precise surgical technique [2, 20, 21]. Careful pre-operative planning, identification of the location of the deformity and accurate surgical technique are mandatory to obtain those good results. Unfortunately, previous studies demonstrated low accuracy for the targeted alignment in conventional OWHTO, with over 30% of outliers from the accuracy range [11]. Furthermore, the association between an over- or under-correction and poor long-term outcomes has been demonstrated [22], and the same has been reported for the modification of the tibial slope, which should be avoided [23]. For all these reasons, a more precise instrumentation, as the one proposed in this study, can be useful in OWHTO.

Some studies described the accuracy of other type of patient-specific instrumentations. Donnez et al evaluated the accuracy of the PSCG in 10 cadavers using a CT scan protocol like the one described in this study. The authors concluded about a high accuracy of the system, with Pearson’s correlation coefficient values similar to those obtained in this study [14]. Miao et al evaluated the accuracy of an angular spacer on 8 specimens, with no significant differences in planned and post-operative MPTA [17]. However, CT scan measurements were performed separately and not superimposed, which may induce doubts in the validity of the comparison. Thanks to the registration process performed in this study, all the measurements were aligned on the same axis, allowing the surgeons to evaluate only the real correction achieved.

There is a paucity of published clinical studies on PSCG’s outcomes [24, 25]. Particularly, Chaouche et al reported about the safety and accuracy of PSCG in 100 OWHTO. The authors concluded about high accuracy of the system also in vivo, with good positioning of the cutting guide in all cases, and an overall complication rate similar to the one reported with the conventional technique [26]. Oher authors similarly concluded on good outcomes and better accuracy of PSCG compared to conventional technique in OWHTO [27]. Finally, in a recent systematic review including 14 studies, Aman et al confirmed good in vivo accuracy with PSCG in both distal femoral and proximal tibia osteotomies, with an outlier rate from the target alignment ranging between 0% and 25% [28]. More recently different studies have been published on the accuracy of PSCG in more complex osteotomies (i.e. double level osteotomy), with good results [29–31]. However, Abdelhameed et al in their series of 91 osteotomies concluded that conventional technique are as accurate as PSCG if performed by an experienced surgeon [32].

Different intra-operative methods to control alignment correction during OWHTO have been proposed other than PSCG, including Computer Assisted Surgery (CAS). Some clinical studies confirmed a higher accuracy for computer navigated OWHTO compared to conventional technique, with still 22% of outliers to the target alignment [11, 33]. However, Hasegawa et al in their clinical study concluded that the Joint Line Convergence Angle (JLCA) was significantly higher in the outlier group, thus underlying a possible defect in pre-operative planning. Furthermore, the same authors concluded that despite the high rate of outliers to the target coronal alignment (18%), mid-term outcomes were still excellent in computer-assisted OWHTO [13].

Another possible problem the surgeons may face during an OWHTO is the hinge fracture, which occurs in 20-30% of the cases, and it is strictly associated to the amount of gap opening [18]. No lateral hinge fractures were detected in this study at the post-operative CT scan, despite the poor bone quality of specimens compared to healthy, vital bone. A careful preoperative planning and surgical technique are surely key factors to reduce the rate of hinge fractures. The PSCG, together with the 3D pre-operative planning, may help surgeons in reducing the rate of hinge fracture, because the position of the hinge and the depth of the osteotomy are carefully assessed during preoperative planning and then reproduced in vivo with the support of the PSC guides.

One of the disadvantages of PSCG is the need for a pre-operative CT scan, which is not performed routinely. However, this technique may reduce the number of intra-operative fluoroscopic images required. Jacquet et al in their clinical study underlined that the average number of fluoroscopic images in OWHTO assisted with a PSCG is around 4 after a learning curve of 9 cases [26].

This study has some limitations. First the small number of specimen and surgeries (6 specimens, 12 cases), even if the power analysis demonstrated that 7 cases were enough to demonstrate statistically significant differences. Furthermore, the specimens did not have a significant varus deformity of the proximal tibia to be considered candidate for an OWHTO. However, two different amounts of deformity correction were simulated (6° and 10°) to be consistent with the clinical practice, and the aim of the study was to assess the accuracy of the system independently from the type of deformity to be corrected. Another limitation is that the pre-operative CT scan is performed in a not weight-bearing situation, which is very different from the “standard” clinical setting, with the planning performed on long-leg, weight-bearing X-rays. Considering the nature of this study (cadaveric study), simulating a weight-bearing condition was not possible. However, in the clinical setting a long-leg, weight-bearing x-rays can be added to the CT scan for a more precise pre-operative planning. Lastly, the reverse engineering process may be limited by the lack of screws, which may influence the precision of the personalized guide.

## Conclusion

OWHTO is a widely performed procedure, with good outcomes if a careful pre-operative planning and accurate surgery are performed. Particularly, high accuracy on both coronal and sagittal plane are mandatory to achieve good outcomes. This study shows good accuracy for 3D pre-operative planning and PSCG in OWHTO, on both coronal and sagittal plane.

## Data Availability

Data are available at the corresponding author.

## Declarations

**Ethics approval and consent to participate**

No ethical approval or informed consent was required.
